# Publisher Correction: Universal probabilistic programming offers a powerful approach to statistical phylogenetics

**DOI:** 10.1038/s42003-021-01922-8

**Published:** 2021-03-15

**Authors:** Fredrik Ronquist, Jan Kudlicka, Viktor Senderov, Johannes Borgström, Nicolas Lartillot, Daniel Lundén, Lawrence Murray, Thomas B. Schön, David Broman

**Affiliations:** 1 Department of Bioinformatics and Genetics, Swedish Museum of Natural History, Stockholm, Sweden; 2 Department of Information Technology, Uppsala University, Uppsala, Sweden; 3 Laboratoire de Biométrie et Biologie Evolutive, UMR CNRS 5558, Université Claude Bernard Lyon 1, Villeurbanne, France; 4 Department of Computer Science, KTH Royal Institute of Technology, Stockholm, Sweden; 5 Uber AI, San Francisco, CA USA

**Keywords:** Phylogenetics, Phylogeny

Correction to: *Communications Biology* 10.1038/s42003-021-01753-7, published online 24 February 2021.

Figures 1 and 2 and their associated captions were missing in the original PDF version of the Article. The online version of the Article was not affected. The errors have been corrected in the PDF version of the Article.

