# Supplementary material for: Universal probabilistic programming offers a powerful approach to statistical phylogenetics
Source: Commun Biol. 2021 Feb 24;4:244. doi: 10.1038/s42003-021-01753-7 (PMC7904853; doi:10.1038/s42003-021-01753-7)
Supplement: Supplementary file 3 — Reporting Summary [file 42003_2021_1753_MOESM3_ESM.pdf]

nature research

Corresponding author(s): Fredrik Ronquist

Last updated by author(s): Jul 28, 2020

Reporting Summary

Nature Research wishes to improve the reproducibility of the work that we publish. This form provides structure for consistency and transparency in reporting. For further information on Nature Research policies, see our Editorial Policies and the Editorial Policy Checklist.

Statistics

For all statistical analyses, confirm that the following items are present in the figure legend, table legend, main text, or Methods section.

n/a	Confirmed	
☒	☐	The exact sample size (*n*) for each experimental group/condition, given as a discrete number and unit of measurement
☒	☐	A statement on whether measurements were taken from distinct samples or whether the same sample was measured repeatedly
☐	☒	The statistical test(s) used AND whether they are one- or two-sided<br>*Only common tests should be described solely by name; describe more complex techniques in the Methods section.*
☐	☒	A description of all covariates tested
☐	☒	A description of any assumptions or corrections, such as tests of normality and adjustment for multiple comparisons
☐	☒	A full description of the statistical parameters including central tendency (e.g. means) or other basic estimates (e.g. regression coefficient) AND variation (e.g. standard deviation) or associated estimates of uncertainty (e.g. confidence intervals)
☒	☐	For null hypothesis testing, the test statistic (e.g. F, t, r) with confidence intervals, effect sizes, degrees of freedom and P value noted<br>*Give P values as exact values whenever suitable.*
☐	☒	For Bayesian analysis, information on the choice of priors and Markov chain Monte Carlo settings
☐	☒	For hierarchical and complex designs, identification of the appropriate level for tests and full reporting of outcomes
☐	☒	Estimates of effect sizes (e.g. Cohen's *d*, Pearson's *r*), indicating how they were calculated

Our web collection on statistics for biologists contains articles on many of the points above.

Software and code

Policy information about availability of computer code

Data collection	No software was used for data collection; our empirical analyses are based on previously published data.
Data analysis	All code developed for the paper is available from https://github.com/phyppl/probabilistic-programming. We also used WebPPL version 0.9.15 (available from https://github.com/probmods/webppl), Node version 12.13.1 (available from https://nodejs.org/en/) and Birch (development version of June 12, 2020; available from https://birch-lang.org/).

For manuscripts utilizing custom algorithms or software that are central to the research but not yet described in published literature, software must be made available to editors and reviewers. We strongly encourage code deposition in a community repository (e.g. GitHub). See the Nature Research guidelines for submitting code & software for further information.

Data

Policy information about availability of data

All manuscripts must include a data availability statement. This statement should provide the following information, where applicable:

- Accession codes, unique identifiers, or web links for publicly available datasets
- A list of figures that have associated raw data
- A description of any restrictions on data availability

The data used to compare the diversification models, together with full literature references, can be found at https://github.com/phyppl/probabilistic-programming, under the directory data.

Field-specific reporting

Please select the one below that is the best fit for your research. If you are not sure, read the appropriate sections before making your selection.

☐ Life sciences ☐ Behavioural & social sciences ☒ Ecological, evolutionary & environmental sciences

For a reference copy of the document with all sections, see nature.com/documents/nr-reporting-summary-flat.pdf

Ecological, evolutionary & environmental sciences study design

All studies must disclose on these points even when the disclosure is negative.

Study description	We develop methods for analysis of statistical phylogenetics problems, in particular phylogenetic diversification models. The methods are applied to previously published data on phylogenetic relationships among birds.
Research sample	We reanalyze the bird phylogeny data from Maliet et al (2017; Nature Ecology & Evolution 3: 1086–1092), originally published by Jetz et al (2012; Nature 491: 444-448).
Sampling strategy	The original paper attempted to sample as many as possible of the known bird species. Different approaches to the modeling of the sampling procedure are discussed in the paper, and the assumptions made in the main analyses are justified.
Data collection	No original data were collected for the study.
Timing and spatial scale	No original data were collected for the study.
Data exclusions	We excluded two clades with negative branch lengths (of 42 in total), as it was unclear how to resolve these errors. This is clearly stated in the paper.
Reproducibility	We used extensive tests to verify the correctness of our analytical software, as documented in the Supplementary information. We also used 500 replicates of each analysis to directly estimate the reproducibility of our empirical results.
Randomization	The study does not include steps where randomization of group allocations would have been relevant.
Blinding	No original data were collected for the study, nor did we use analysis methods where blinding would have been relevant.

Did the study involve field work? ☐ Yes ☒ No

Reporting for specific materials, systems and methods

We require information from authors about some types of materials, experimental systems and methods used in many studies. Here, indicate whether each material, system or method listed is relevant to your study. If you are not sure if a list item applies to your research, read the appropriate section before selecting a response.

Materials & experimental systems

n/a	Involved in the study	
☒	☐	Antibodies
☒	☐	Eukaryotic cell lines
☒	☐	Palaeontology and archaeology
☒	☐	Animals and other organisms
☒	☐	Human research participants
☒	☐	Clinical data
☒	☐	Dual use research of concern

Methods

n/a	Involved in the study	
☒	☐	ChIP-seq
☒	☐	Flow cytometry
☒	☐	MRI-based neuroimaging
